# Whole genome comparative studies between chicken and turkey and their implications for avian genome evolution

**DOI:** 10.1186/1471-2164-9-168

**Published:** 2008-04-14

**Authors:** Darren K Griffin, Lindsay B Robertson, Helen G Tempest, Alain Vignal, Valérie Fillon, Richard PMA Crooijmans, Martien AM Groenen, Svetlana Deryusheva, Elena Gaginskaya, Wilfrid Carré, David Waddington, Richard Talbot, Martin Völker, Julio S Masabanda, Dave W Burt

**Affiliations:** 1 Department of Biosciences, University of Kent, Canterbury, Kent, CT2 7NJ, UK; 2 Laboratoire de Génétique Cellulaire, Centre INRA de Toulouse, BP 27 Auzeville, 31326 Castanet Tolosan, France; 3 Animal Breeding and Genomics Centre, Wageningen University, Marijkeweg 40, 6709 PG Wageningen, The Netherlands; 4 Biological Research Institute, Saint-Petersburg State University, Oranienbaumskoie sch. 2, Stary Peterhof, Saint-Petersburg, 198504, Russia; 5 Dept. of Genomics & Bioinformatics, Roslin Institute/Edinburgh University, Midlothian, EH25 9PS, UK; 6 Current address : Bridge Genoma, The London Bioscience Innovation Centre, 2 Royal College Street London. NW1 0NH, UK; 7 Current address : Department of Medical Genetics, University of Calgary, 3330 Hospital Dr, NW, Calgary, AB, T2N 4N1, Canada; 8 Current address : ID LABS(tm) Inc., 100 Collip Circle, Unit 117 London, Ontario, N6G 4X8, Canada; 9 Current address : Dept Genomics and Genetics The Roslin Institute and Royal (Dick) School of Veterinary Studies Midlothian EH25 9PS, UK

## Abstract

**Background:**

Comparative genomics is a powerful means of establishing inter-specific relationships between gene function/location and allows insight into genomic rearrangements, conservation and evolutionary phylogeny. The availability of the complete sequence of the chicken genome has initiated the development of detailed genomic information in other birds including turkey, an agriculturally important species where mapping has hitherto focused on linkage with limited physical information. No molecular study has yet examined conservation of avian microchromosomes, nor differences in copy number variants (CNVs) between birds.

**Results:**

We present a detailed comparative cytogenetic map between chicken and turkey based on reciprocal chromosome painting and mapping of 338 chicken BACs to turkey metaphases. Two inter-chromosomal changes (both involving centromeres) and three pericentric inversions have been identified between chicken and turkey; and array CGH identified 16 inter-specific CNVs.

**Conclusion:**

This is the first study to combine the modalities of zoo-FISH and array CGH between different avian species. The first insight into the conservation of microchromosomes, the first comparative cytogenetic map of any bird and the first appraisal of CNVs between birds is provided. Results suggest that avian genomes have remained relatively stable during evolution compared to mammalian equivalents.

## Background

Comparative genomics is a powerful means for establishing relationships between gene function and location in a range of organisms. Moreover it allows insight into large-scale genomic rearrangements, conservation of functional elements and tracing of evolutionary phylogenies through the examination of both closely and distantly related species. The completion of the chicken (*Gallus gallus*, GGA) genome [1] and its associated resources provide the basis for the rapid development of detailed genomic information, potentially in all other birds. The most powerful strategies combine *in-silico *and experimental approaches, e.g. sequence comparison, cross-species fluorescent *in-situ *hybridization (zoo-FISH) [2,3] and, more recently, the use of whole-genome tiling path microarrays for cross-species array comparative genomic hybridization (array CGH) [4-8]. Such a combination of modalities provides information on gross genomic rearrangements, gene gains/losses, copy number variation and gene order.

Following the completion of the chicken genome sequence assembly [1], one of the most obvious targets for comparative genomics in birds is the turkey *(Meleagris gallopavo*, MGA). Turkey is an agriculturally important species accounting for over 4.5 million tonnes of meat consumed per year worldwide, with the obvious cultural associations such as Christmas and Thanksgiving. Genetic mapping efforts in the turkey have focused on linkage mapping [9-13] and the physical information available is very limited. With 2n = 78 in the chicken and 2n = 80 in the turkey, about 10 pairs of macro- and 28–30 pairs of microchromosomes in both species, the karyotypes of chicken and turkey are quite similar to the hypothetical ancestral Galliform karyotype [14]. Chromosome banding and zoo-FISH with chromosome paints for chicken chromosomes GGA1–9 and Z in a range of Galliform species have suggested that chicken and turkey karyotypes are distinguished by at least two interchromosomal rearrangements [15,16]. That is, the orthologues of chicken chromosomes GGA2 and 4 are represented by turkey chromosomes MGA3 & 6, and 4 & 9 respectively [14]. Comparisons of a series of other Galliformes suggest that GGA2 is the ancestral form (the acrocentric MGA3 and MGA6 suggesting a breakpoint in the short arm just above the centromere). By contrast, the most parsimonious explanation for the formation of the sub-metacentric GGA4 suggests a fusion of an ancestral acrocentric chromosome 4 with a smaller chromosome [14]. This fusion model is supported by sequence evidence which suggests that GGA4p retains the properties (e.g. high gene density, high recombination frequency) of the smaller chromosome it once was [1]. The chromosomal break- and fusion points involved in these rearrangements however have not been characterized in detail, nor has gene order on macrochromosomes; moreover no molecular evidence has yet been generated regarding synteny between microchromosomes though simple chromosome counts suggest extensive conservation.

To the best of our knowledge, no study has examined inter-specific differences in copy number variants (CNVs) between birds. CNVs are defined as copy number changes involving DNA fragments that are ~1 kb or larger [17], with the exception of insertions or deletions of transposable elements [18]. Recent high-resolution, high-throughput techniques for genomic analysis such as array CGH and quantitative (real-time) PCR as well as *in-silico *approaches have revealed a significant contribution of CNVs to human genetic variation [18,19], and studies in humans and other primates have suggested an important role for CNVs in disease-related as well as normal phenotypic variation [18,19] and in evolutionary adaptation [4-6,20-22]. However, the paucity of data and the almost exclusive focus on primates preclude any general conclusions about the significance of CNVs in phenotypic variation and evolution; data from other species is therefore essential.

In this paper, by examination of chicken and turkey genomes for chromosomal and CNV differences we test the hypothesis that Galliform genomes have remained relatively stable during ~28 million years of evolution compared to an equivalent period in mammals. To date, this information has been limited to zoo-FISH of chicken chromosome paints on other species and partial karyotypes but these early studies do suggest relative stability of the avian genome compared to the mammalian one. We thus present a detailed comparative cytogenetic map of the turkey based on reciprocal zoo-FISH with chicken and turkey macrochromosome paints, zoo-FISH with chicken microchromosome paints and single/dual color FISH mapping of more than 300 chicken BACs to turkey metaphases. In addition, we provide a molecular characterization of inter- and intra-chromosomal rearrangements by FISH mapping of BACs to chicken metaphase chromosomes, painting of chicken lampbrush chromosomes with turkey chromosome paints and hybridization of turkey chromosome paints onto a chicken whole genome tiling array. Finally, we present comparative data on CNVs in chicken and turkey, which constitute the first array CGH-based data set on inter-specific differences of CNVs in birds.

## Results

### Chromosome painting

**Figure 1:**
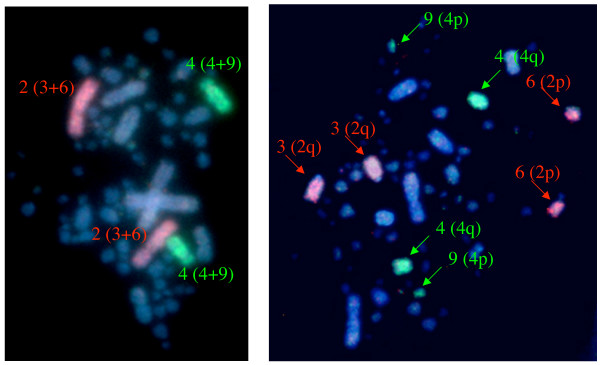
**Chromosome paints for chicken chromosomes GGA2 (red) and GGA4 (green)**. a) On a chicken metaphase (chromosome numbers are labeled with turkey (MGA) orthologues in brackets). b) On a turkey metaphase (chromosome numbers are labeled with arrows and chicken (GGA) orthologues are indicated in brackets).

**Figure 2:**
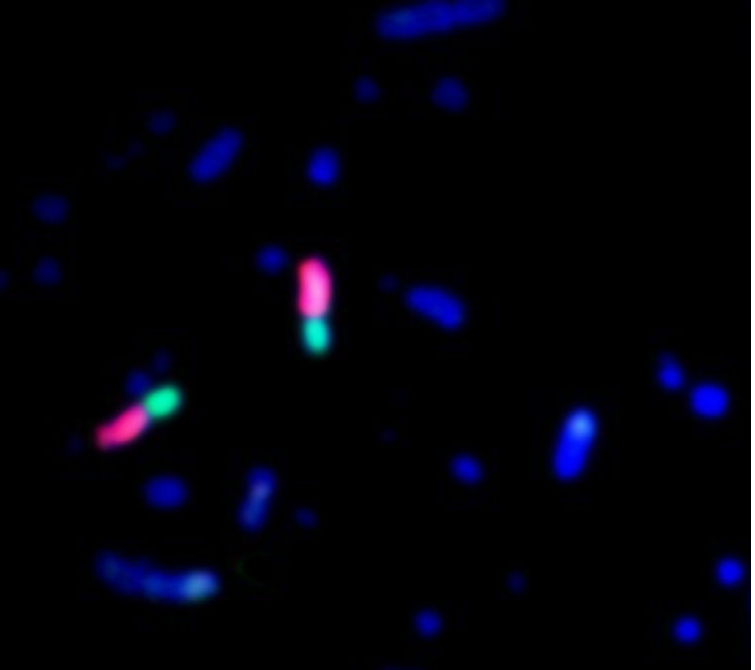
Chromosome paints for turkey chromosomes MGA3 (red) and MGA6 (green) hybridized on chicken (GGA) metaphase chromosomes suggesting that the breakpoint is centromeric.

**Figure 3:**
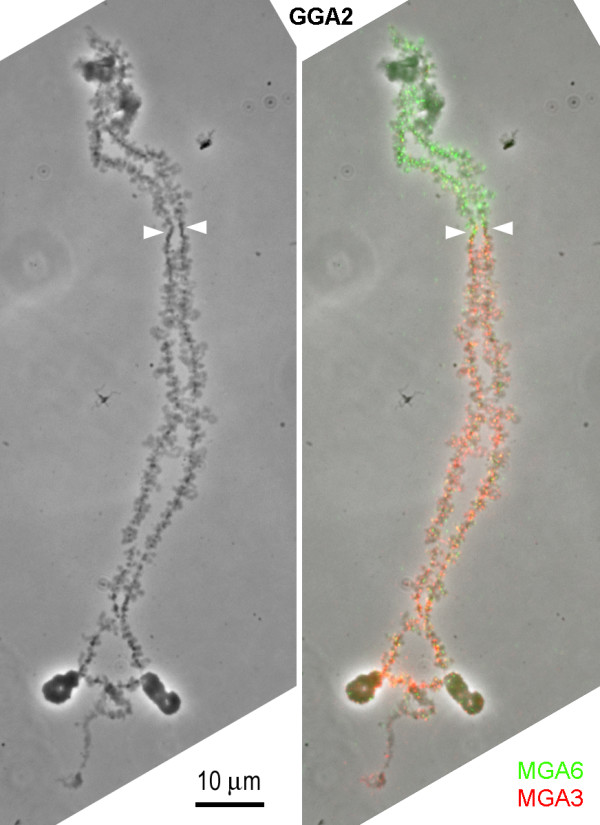
**Chromosome paints for turkey chromosomes MGA3 (red) and MGA6 (green) hybridized on chicken lampbrush chromosome GGA2 confirming that the breakpoint is centromeric**. The left-hand image is a phase contrast picture, the right-hand image is the same micrograph with the chromosome paint hybridizations superimposed. The centromere is arrowed.

Cross-species hybridization of chicken chromosomes GGA1–13, 18–21 and 24–27 plus the Z chromosome to turkey metaphases confirmed two inter-chromosomal rearrangements between the two species. The chromosome paint for GGA2 hybridized to both turkey chromosomes MGA3 and 6, while the chromosome paint for GGA4 hybridized to MGA4 and a smaller chromosome which banding and ideogram analysis [16] suggest to be MGA9 (Figure 1). Reciprocal painting of chicken chromosomes with chromosome paints for MGA1–9 and Z confirmed these results and were consistent with both inter-chromosomal rearrangements involving the centromere of GGA2 and 4 respectively (Figure 2). Orthology of GGA4p and MGA9 was also confirmed by comparative FISH mapping of chicken BACs (see below). Further evidence of centromeric involvement came from the hybridization of chromosome paints for MGA3, 4 and 6 onto chicken extended lampbrush chromosomes (illustrated for MGA3 and 6 in Figure 3); hybridization of the MGA9 paint was not successful on lampbrush chromosomes. The chromosome paint for GGA25 hybridized strongly to metaphases of both species, however the size of the chromosome painted appeared much larger in turkey than in chicken. Numerous attempts at cross-species painting for the remainder of the microchromosomes (GGA28–38) were not successful and chromosome paints were not available for chromosomes GGA14–17, 22 and 23.

### Single and dual color FISH mapping of BACs

**Figure 4:**
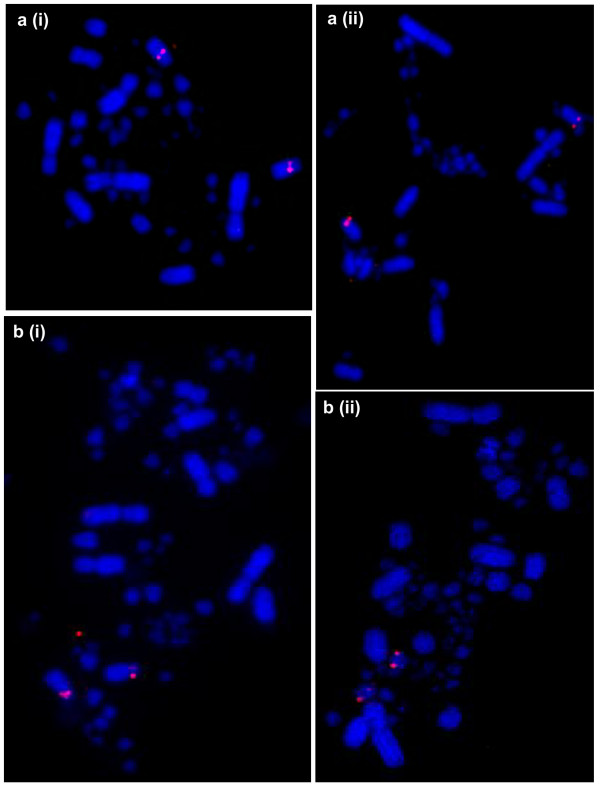
**BACs flanking the centromere of chicken chromosome 4**. a) BAC43O01 mapping to i) chicken 4q; and ii) turkey 4. b) BAC44F19 mapping to i) chicken 4p; and ii) turkey 9.

**Figure 5:**
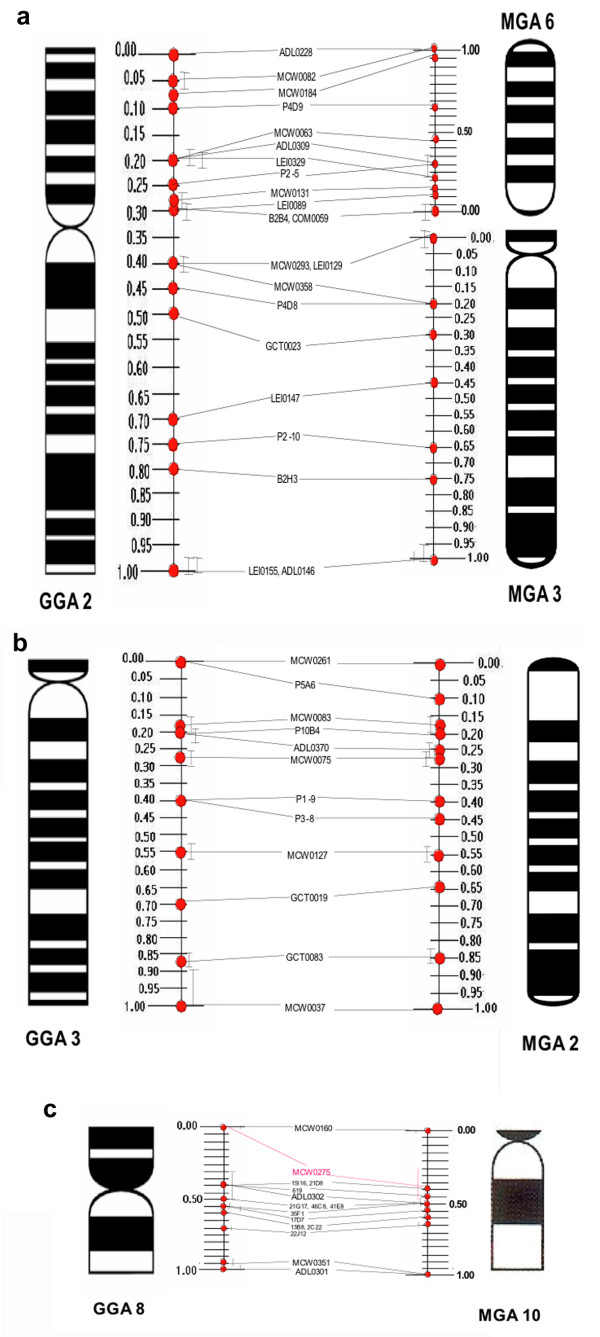
**Ideograms indicating relative hybridization positions of BACs and FLpter values**. Signal positions and FLpter values are indicated for. a – Chicken chromosome 2 (GGA2) and turkey chromosomes 3 and 6 (MGA3+6). b – Chicken chromosome 3 (GGA3) and turkey chromosome 2 (MGA2). c – Chicken chromosome 8 (GGA8) and turkey chromosome 10 (MGA10). The hybridization positions of BACs were determined by measuring the fractional length of the signal position from the p-terminus (see Methods).

Using single and dual color FISH we successfully mapped 338 BACs in both chicken and turkey (e.g. Figure 4), approximately 70% of the BACs mapped in chicken also mapped to turkey. One or more chicken BACs from GGA1–28 and Z (with the exception of GGA21, 22 and 25) all successfully hybridized to turkey chromosomes. These experiments confirmed the chromosome painting results and, in addition, suggested that there were no further inter-chromosomal rearrangements. Three intra-chromosomal rearrangements nevertheless were detected: The centric nature of the breakpoint that led to the evolution of MGA3 and MGA6 and the presence of a small short arm on MGA3 which contains the BACs identified by markers MCW0293 and LEI0129 (both of which hybridize below the centromere in chicken) is consistent with a pericentric inversion (Figure 5a). Moreover, these two BACs co-localize with BAC MCW0358 on GGA2 but are approximately 20% of the chromosome further away in MGA3 (Figure 5a), again suggesting that a pericentric inversion is the likely mechanism. For GGA3 and its orthologue MGA2, the presence of BAC P5A6 above the centromere in chicken but around one tenth of the way down chromosome 2 in turkey (and below the centromere) again, provides evidence of a pericentric inversion (Figure 5b). Finally the presence of BAC MCW0275 near the middle of MGA10 (an acrocentric chromosome) and towards the telomere of GGA8 (a metacentric chromosome) gave clear evidence of a third pericentric inversion (Figure 5c). The FISH mapping data were used to construct a cytogenetic map of the turkey, which have been uploaded to a publicly available database on the ArkDB browser [23]. Chromosome painting and BAC mapping data for the microchromosomes are summarized in Table 1.

**Table 1:** Chicken BAC clones and chromosome paints mapped successfully to both chicken (GGA) and turkey (MGA) microchromosomes. Up to and including GGA28, only GGA22 has neither a BAC nor a paint successfully hybridizing to both species; for GGA16 only one BAC could be hybridised to both species. The remainder all have at least 2 BACs and/or a paint tagging the chromosome.

**Chicken chromosome number (GGA)**	**Turkey orthologue number (MGA)**	**BAC clone name**	**Chicken genetic marker**	**Chromosome paint successful on both species?**
9	11	BW009J23	ROS0078	Yes
9	11	BW027D19	ADL0191	Yes
9	11	BW010E13	MCW0017	Yes
9	11	BW089K16	ABR0018	Yes
9	11	BW014A21	MCW0134	Yes
10	12	BW009C07	LEI0333	Yes
10	12	BW008K20	MCW0228	Yes
10	12	BW018B17	COM0101	Yes
10	12	BW046J07	MCW0194	Yes
10	12	BW015I08	ADL0272	Yes
10	12	BW092I14	ADL0231	Yes
10	12	BW018I03	MCW0035	Yes
10	12	BW085M07	ADL0120	Yes
10	12	BW014B17	ADL0106	Yes
10	12	BW008G10	MCW0132	Yes
10	12	BW006H24	IGF1R	Yes
10	12	BW089E07	GNRHR	Yes
10	12	BW026A13	RPL4	Yes
10	12	BW055K19	LEI0112	Yes
10	12	BW035L19	MCW0003	Yes
10	12	BW012O09	ABR0012	Yes
10	12	BW098C19	ADL0112	Yes
11	13	BW035F15	LEI0143	Yes
11	13	BW012F03	LEI00110	Yes
11	13	BW010A05	ADL0232	Yes
11	13	BW029L10	MCW0097	Yes
11	13	BW087P13	ADL0123	Yes
11	13	BW003B03	ADL0287	Yes
11	13	BW084D24	ADL0041	Yes
11	13	BW054E23	ADL0308	Yes
11	13	BW053F11	ABR0037	Yes
12	14	BW017B16	ADL0372	Yes
12	14	BW033L02	ADL0240	Yes
12	14	BW047D10	LEI0099	Yes
12	14	BW067B16	MCW0198	Yes
12	14	BW011C21	MCW0332	Yes
13	15	BW014G12	MCW0244	Yes
13	15	BW018H02	MCW0213	Yes
13	15	BW027P20	MSX2	Yes
13	15	BW008N24	SDX1	Yes
13	15	BW025H18	CAMLG	Yes
14	16	BW019L14	CTG7070	No
14	16	BW026C02	MCW0136	No
14	16	BW043B20	MCW0296	No
14	16	BW026J16	GCT0908	No
14	16	BW053L23	ADL0200	No
14	16	BW014I20	LEI0098B	No
14	16	BW002J02	MCW0123	No
14	16	BW060O01	GCT0903	No
14	16	BW068N21	ROS0005	No
15	17	BW109B14	MCW0031	No
15	17	BW003B07	ADL0206	No
15	17	BW010L01	LEI0083	No
15	17	BW017C11	LEI0120	No
15	17	BW023P01	ADL0039A	No
15	17	BW107C20	MCW0231	No
15	17	BW047O08	GCT0014	No
15	17	BW030E18	MCW0080	No
15	17	BW007G01	MCW0211	No
16	18	BW065G09	MCW0371	No
17	19	BW117D07	HSP5A	No
17	19	BW040G23	ADL0149	No
17	19	BW023I07	MCW0330	No
17	19	BW013I13	MCW0151	No
17	19	BW020L20	LEI0342	No
17	19	BW032N22	ADL0202	No
18	20	BW122G20	HUJ0010	Yes
18	20	BW019B13	MCW0045	Yes
18	20	BW027I07	ROS0022	Yes
18	20	BW034F23	ADL0290	Yes
18	20	BW000O20	ADL0184	Yes
18	20	BW014H23	ROS0027	Yes
18	20	BW001D02	MCW0219	Yes
19	21	BW086B08	LEI0330	Yes
19	21	BW055M22	SCW0024	Yes
20	22	BW020P15	GCT0039	Yes
20	22	BW022L03	ADL0193	Yes
20	22	BW084E10	ADL0034	Yes
20	22	BW010A11	FZFsts1	Yes
21	23	No BACS		Yes
22	24	No BACS		No
23	25	BW025H08	ADL0262	No
23	25	BW096F24	LEI0339	No
23	25	BW010D11	ADL0289	No
23	25	BW029E23	LEI0102	No
23	25	BW028L18	LEI0090	No
24	26	BW013J20	ROS0113a	Yes
24	26	BW008L04	ROS0123	Yes
24	26	BW020E08	APOA1sts	Yes
24	26	BW032F12	LEI0069	Yes
25	27	No BACS		Yes
26	28	BW056B11	MCW0262	Yes
26	28	BW005G11	MCW0286	Yes
26	28	BW077M23	MCW0209	Yes
26	28	BW028I01	MCW0069	Yes
26	28	BW019A06	ABR0006	Yes
27	29	BW029K01	CTG2535	Yes
27	29	BW028K09	MCW0233	Yes
27	29	BW009E08	MCW0146	Yes
27	29	BW008C15	MCW0328	Yes
27	29	BW003A13	GCT0022	Yes
28	30	BW036G05	LEI0135	No
28	30	BW017C23	ROS0266	No
28	30	BW024J22	ABR0341	No
28	30	BW005E17	GCT0904	No
28	30	BW029E08	LEI0067A	No
29–38	31–39	No BACS		No

As mentioned above, chromosome painting experiments suggest centromeric breakpoint/fusion points in GGA2 and GGA4. This was confirmed by systematic FISH mapping of a further 108 BACs to GGA2 and 4 and MGA3, 6, 4 and 9. Of these BACs, 51 were on GGA2p and MGA6, 18 were on GGA2q and MGA3, 13 were on GGA4p and MGA9 and 26 were on GGA4q and MGA4. These experiments narrowed down the breakpoint in GGA2 to a region of 1.7 Mb (between BACs bW018L21 and bW018G01) and the fusion point in GGA4 to a region of 2.46 Mb (between BACs bW044F19 and bW043O01 (Figure 4)). Bioinformatic analysis of these BACs in relation to the most recent chicken genome assembly (WUSTL 2.1) is consistent with the presence of the centromere between them in both cases, i.e. they flank nucleotide positions 52,291,242 & 53,791,241 on GGA2, and 19,307,569 & 20,807,568 on GGA4 respectively.

### Turkey chromosome paints on the chicken microarray

**Figure 6:**
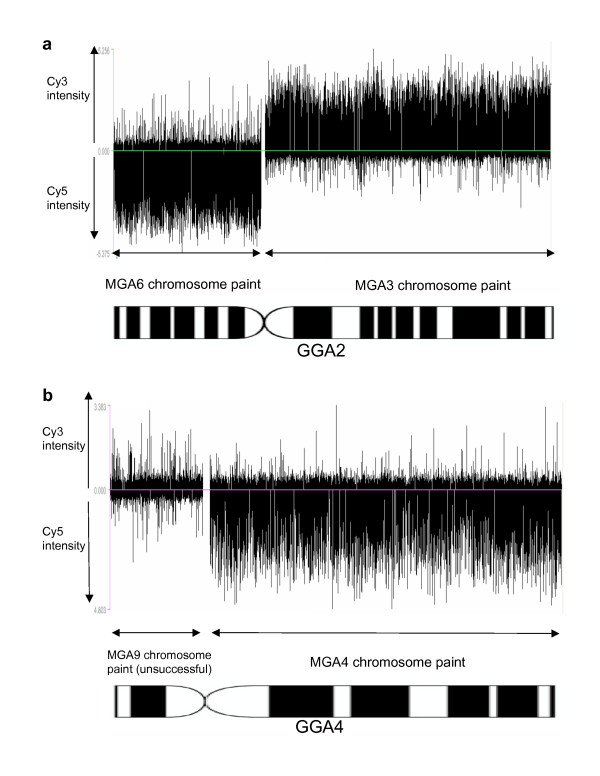
**Microarray experiments using turkey (MGA) chromosome paints on the NimbleGen microarray**. a. Using chromosome paints for MGA3 (Cy3) and MGA6 (Cy5). b. Using chromosome paints for MGA9 (Cy3) and MGA4 (Cy5). Points above the midline represent signals in the Cy3 range; points below the midline represent signals in the Cy5 range. The results indicate failure of hybridization for the MGA9 chromosome paint but successful hybridization for the other three. All results are consistent with centromeric breakpoint/fusion points.

Hybridization of DNA derived from flow-sorted turkey chromosomes MGA3 and 6 (orthologous to GGA2) and MGA4 and 9 (orthologous to GGA4) to the NimbleGen chicken whole genome tiling array further confirmed the centromeric nature of the GGA2: MGA [3+6] breakpoint and the MGA [4+9]: GGA4 fusion point (Figure 5). Hybridization of MGA3 and 6 gave positive hybridization signals on GGA2 between positions 52,287,000 and 53,795,000 (Figure 6a). This is the position of the centromere on GGA2 estimated to be located between positions nucleotides 52,291,242 and 53,791,241 (WUSTL 2.1). Hybridization of MGA4 gave positive hybridization signals on GGA4 beyond positions 20,808,000 (Figure 6b). This is the position of the centromere on GGA4 estimated to be located between positions nucleotides 19,307,569 and 20,807,568 (WUSTL 2.1). Hybridization using the MGA 9 paint failed to show any positive signal. Thus the results of the CGH approach were consistent with those of reciprocal chromosome painting (on both metaphase and lampbrush chromosomes, Figures 2 and 3) and those of the systematic BAC mapping.

### Sequence analysis of chicken chromosome arms

**Figure 7:**
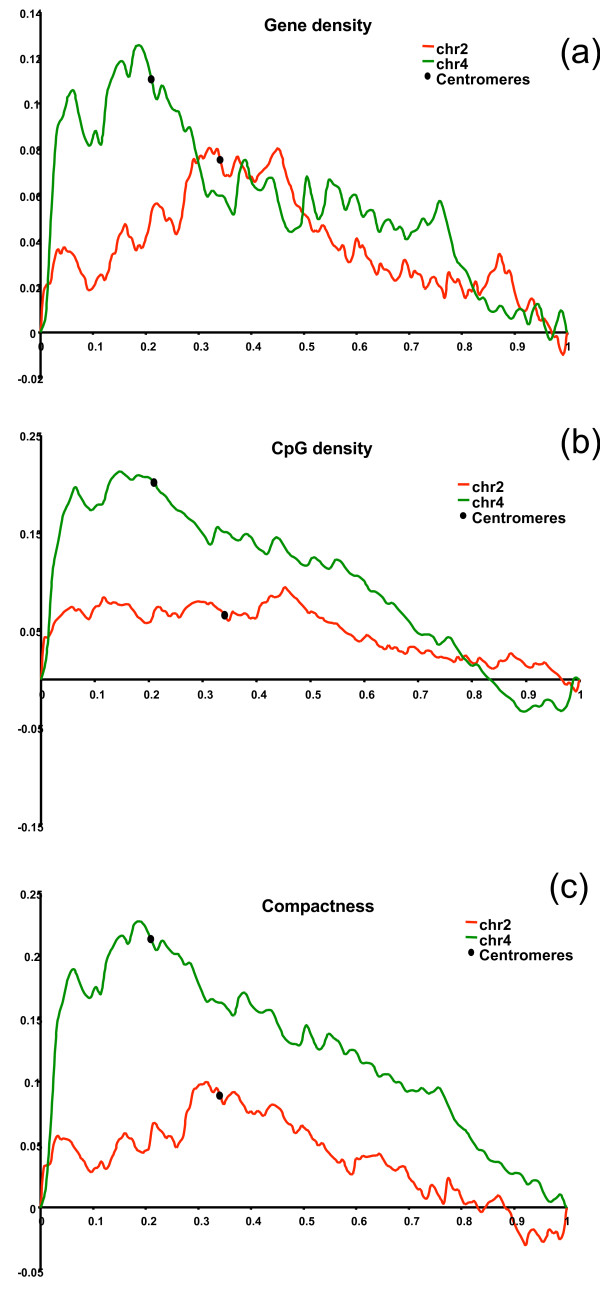
Gene density (a), CpG density (b) and compactness (c) of chicken chromosomes GGA2 and GGA4.

Previous sequence analysis of GGA4 [1] suggested that GGA4p retains the sequence properties (e.g. high gene density, high recombination frequency) of the microchromosome it once was. An in depth analysis of the sequence characteristics of the chromosome arms for both GGA2 and GGA4 is presented in Table 2 and in the cumulative plots in Figure 7. The analysis of gene density shows clearly that the number of genes per length unit is higher in the p-arm of chromosome 4 than in the q-arm. This higher gene density is also revealed by a higher CpG-island density and greater gene compactness. In addition the shorter arm is more GC-rich and has a higher rate of genetic recombination. In contrast, the characteristics of GGA2p and GGA2q are very similar.

**Table 2:** Sequence composition of chicken chromosomes GGA2 and GGA4.

**Parameter**	**GGA2p**	**GGA2q**	**GGA4p**	**GGA4q**
Physical length (bp)	52,291,242	94,991,177	19,307,568	73,422,834
Recombination rate (cM/Mb)	3.09	2.54	4.48	2.2
G+C content of chromosomes (%)	40.44 ± 3.67	39.39 ± 2.95	42.73 ± 5.77	39.32 ± 3.45
Gene Density (genes/Mb)	12.01	8.69	19.53	10.87
CpG density (Islands/Mb)	39.38	29.68	87.12	32.78
Repeats (%)	6.44%	12.14%	5.43%	8.05%
Introns length (bp/gene)	38,870 ± 65,112	39,407 ± 66,942	19,542 ± 40,675	33,587 ± 53,907
G + C content of introns (%)	42.38 ± 7.82	41.06 ± 6.33	47.47 ± 8.28	41.84 ± 6.84
Exons length (bp/gene)	1,967 ± 1808	1,813 ± 1720	1,750 ± 1340	1,941 ± 1629
G + C content of exons (%)	47.77 ± 7.63	46.28 ± 7.07	51.36 ± 7.13	46.91 ± 6.72

### Array CGH experiments

Hybridization of turkey whole genomic DNA on the NimbleGen chicken tiling path microarray revealed evidence of 16 CNVs (Table 3). The mean and median lengths of CNVs were 179 kb and 90 kb, respectively and ranged from 30 kb to 900 kb. From this analysis it is not clear whether these are insertions or deletions but 15/16 showed a "loss" of signal in the turkey. These may also represent regions of poor sequence homology and thus regions that have undergone accelerated sequence divergence. We compared the location of these CNVs with the location of CNV polymorphisms in the broiler and layer lines of chickens (Table 3). In all cases the genome of Red Jungle Fowl sequence [1] was used as a reference DNA. Comparisons between Red Jungle Fowl and broiler/layer lines detected 12 CNVs with 8 CNVs/individual distributed on both macro- and micro-chromosomes. The mean and median lengths of CNVs were 127 kb and 90 kb, respectively (ranging from 30 to 300 kb). These lengths are similar to that found for CNVs found in human [24] but fewer in number with an average of 24/human individual compared to the 8/chicken sample. Comparison of the location of turkey/chicken and chicken/chicken CNVs suggests that 50% of the CNVs are likely to have identical positions. For the purpose of this study, they were designated as "hotspots" if the same CNV appeared more than once in different animals (e.g. in broiler, layer and/or turkey – Table 3). The median length of CNVs is also identical at 90 kb.

**Table 3:** Copy number variants of turkey plus broiler and layer chickens compared to Red Jungle Fowl. Numbers represent fold-changes compared to Red Jungle Fowl (when significantly different). Table also notes whether the CNV is associated with a gene/genes and degree of conservation.

**CNV**	**Chromosome Location**	**Length**	**Fold change of CNV cf, Red Jungle Fowl**			**CNV Hotspot?**	**Genes/ESTs**	**Conserved in Mammals?**	**Conserved in *Xenopus*/Zebrafish?**	**Comments**
							
		**(kb)**	**Broiler**	**Layer**	**Turkey**					
1	chr1:86445000–86475000	30,000			-5.65		yes	no	yes	novel proteins, birds/fish/frogs, Phospholipase A2, a family of secretory and cytosolic enzymes
2	chr1:104475000–104505000	30,000	-1.95				yes	yes	yes	novel protein, birds/fish/frogs
3	chr1:140595000–140625000	30,000	2.26		-5.52	yes	yes	yes	yes	similar to RAN binding protein 2
4	chr1:144225000–144255000	30,000			-3.54		yes	yes	yes	novel proteins/RNAs
5	chr1:165885000–165975000	90,000	-2.59	-2.24			yes	yes	yes	interleukin enhancer binding factor 3
6	chr1:197715000–197745000	30,000			-2.83		yes	no	yes	novel, birds/frogs, similar to Gab2, lipid binding motifs
7	chr10:30000–150000	120,000			-1.82		yes	yes	yes	olfactory receptors
8	chr13:510000–570000	60,000	-2.05				yes	yes	yes	protocadherin cluster
9	chr13:18885000–18903772	18,772			-4.73		yes	yes	yes	novel proteins/RNAs
10	chr16:15000–426425	411,425	-1.48	-2.68	-4.73	yes	yes	yes	yes	MHC
11	chr2:25725000–25785000	60,000		-1.57			no	yes	yes	conserved non-coding region
12	chr2:49185000–49215000	30,000		2.13			yes	no	yes	novel, birds/frogs, T cell receptor, gamma cluster
13	chr2:53925000–54225000	300,000			-1.62		yes	yes	yes	Thiamin pyrophosphokinase 1
14	chr27:30000–930000	900,000			-1.81		yes	yes	yes	gamma filamin protein, mainly due to cluster of feather keratins
15	chr27:165000–255000	90,000		-1.47			yes	no	yes	novel proteins/RNAs birds/fish
16	chr3:113550000–113646334	96,334	2.34	2.30	-2.59	yes	yes	yes	yes	39S ribosomal protein L19, GC-rich sequence DNA-binding factor TCF9-like, novel proteins/RNAs
17	chr4:88935000–89025000	90,000		-2.20	-2.42	yes	yes	no	no	CD8alpha
18	chr6:4695000–4785000	90,000	-1.70		-3.98		yes	yes	yes	Pro-neuregulin-3
19	chr6:11475000–11505000	30,000			-9.91		yes	no	no	novel proteins/RNAs
20	chr7:23625000–23925000	300,000			1.67		yes	yes	yes	many genes, mainly due to TUBA-cluster
21	chrE64:22523–22523	n/d			-1.41		yes	yes	yes	Zinc finger cluster
22	chrZ:71535000–71745000	210,000	3.45	3.38	-7.01	yes	yes	yes	yes	male/female difference, similar to rac GTPase activating protein

Total number of CNV loci cf. Red Jungle Fowl			8	8	16	6				

The location of 21 out of 22 (95.5%) CNVs were associated with genes or "predicted genes." Only in one case (on chromosome 2, position 25,725,000–25,785,000) did there appear to be no genes associated with the CNV. This region however represented a highly conserved non-coding region of unknown function. In 20 out of the 22 CNVs (90.9%) the regions were conserved in chicken, *Xenopus *and zebrafish but, of those 20, only 16 were conserved in mammals. This suggested either loss or rapid sequence divergence in mammalian lineages. Many novel genes appear to be predicted in the non-mammalian lineages which would support gene loss scenario. Cases of rapid divergence are supported by associations with the rapidly evolving proteins of the immune (e.g. MHC and interleukin enhancer binding factor 3) and chemosensory (e.g. olfactory receptors) systems.

## Discussion

### Detailed molecular cytogenetic map of the turkey

To the best of our knowledge, the cytogenetic map of the turkey presented here is the first detailed cytogenetic map of any avian species produced by comparative chromosome painting and FISH mapping of chicken BACs to another species. The availability of a comparative map between turkey and chicken will, first of all, allow the transfer of genetic information directly from chicken to turkey, thus expediting mapping studies in turkey. Secondly it will help to target marker development in turkey through the prediction of new loci. Finally it, and maps of other species, will provide insight into the conservation and function of sequences across avian and other vertebrate species. This data will include coding sequences and also functional non-coding sequences such as telomeres. The map is sufficiently detailed to allow accurate gene prediction in turkey particularly given that the rearrangements between chicken and turkey are relatively few.

### Chromosomal rearrangements between chicken and turkey

Previous studies based on chromosome banding [16] and zoo-FISH with chicken macrochromosome paints [15] suggest that GGA2 represents the ancestral state (reviewed in [14]) whereas GGA4 represents a derived chromosome [14]). This model is supported by sequence analysis which suggests that GGA4p retains the ancestral sequence characteristics of the smaller chromosome it once was i.e. high gene density, compact genes, high CpG-island density, high recombination frequency and GC-richness [1] whereas GGA2 does not.

In the current study further insights are provided than were previously detectable by banding: On GGA2 and its orthologues, banding analysis had previously been consistent with a breakpoint *above *the centromere (since MGA3 clearly has a small p-arm). The evidence provided here however suggests that the breakpoint is centromeric and thus a second mechanism must have been involved in the origin of MGA3p, probably a pericentric inversion.

The fusion point of GGA4, as well as the two other inter-chromosomal rearrangements was largely as predicted from G-banding analysis, i.e. involving the centromeres. Moreover, although a few of the FLpter measurements between the two species led to "crossed lines" (e.g. ABR0047 and GCT0002 in GGA1 and MGA1, MCW0193 and P6C6 in GGA5 and MGA5), all of these were within an acceptable margin of error and thus no evidence of paracentric inversions was found. The locations of BACs on the sequence maps are in most cases derived from the position of the (microsatellite) marker that was used to isolate that particular BAC. Therefore some of these inconsistencies may be explained by an incorrect marker-BAC pair.

For GGA11–21 and GGA23–28 we provide the first evidence suggesting that there are no inter-chromosomal changes between chicken and turkey among this set of microchromosomes. This is in line with results from earlier studies that used dual color FISH mapping of a limited number of BACs to examine synteny of microchromosomes in duck (*Anas platyrhynchos*) [25] and quail (*Coturnix japonica*) [26] and found no evidence for inter-chromosomal rearrangements among microchromosomes. In the present study, only metaphase microchromosomes were examined, which provides limited resolution due to the small size of the microchromosomes; we therefore cannot preclude the existence of intra-chromosomal rearrangements until experiments with very elongated (e.g. lampbrush) chromosomes are performed. Moreover we cannot exclude the existence of small local rearrangements that are beyond the resolution of our mapping methods. For chromosomes 29–38 (the D-group), chromosome painting experiments were largely unsuccessful, we believe this was because these chromosome paints were generated from single templates and were comparatively weak, even on chicken metaphases [27]. The relatively different sizes of GGA25 and its turkey orthologue MGA27 provide evidence for our previous suggestion that this chromosome paint is so bright compared to others largely because the chromosome consists of repeat elements in chicken [27]. The paucity of markers on the sex chromosomes precluded a full analysis of the Z (only 4 markers were examined on the Z chromosome) and any analysis at all on the W chromosome. The position of the centromere on the Z chromosome is different in chicken and turkey, but this most likely results from the accumulation of heterochromatin on the chicken Z [15].

Recent evidence [1] suggests that chicken chromosome 22 (GGA22) differs from the other group B-D chromosomes (chromosomes 11–38) in having a GC content, exon density and repeat density similar to that of the group A chromosomes (1–10 and Z) and may be translocated to chromosome 3 in turkey. Unfortunately, and frustratingly although we had several BAC markers from chicken chromosome 22, none worked successfully across species despite several attempts; we were thus unable to establish whether a further fission or fusion event occurred between the ancestral orthologues of GGA3 and GGA22. It is notable however that the use of the chromosome paint for MGA3 on to chicken chromosomes did not reveal hybridization to a microchromosome in chicken.

The results of the present study therefore confirm that chicken and turkey karyotypes (common ancestor 25–30 MYA) have undergone very few chromosomal rearrangements during evolution and extends this finding to the majority of the microchromosomes. By comparison, humans and new world monkeys (common ancestor 30–35 MYA) have around 10 inter-chromosomal rearrangements; humans and lesser apes (common ancestor 15–20 MYA) have many more [2,3], rats and mice (common ancestor 10–20 MYA) have 14 [2,3]. Although it might be argued that mice and lesser apes are extreme examples of where extensive rearrangements have occurred, the general rate of inter-chromosome rearrangement in mammals is thought to be one every 10 million years [2,3]. If this model held true for birds then we might expect about 5 or 6 differences between chicken and turkey since they diverged 25–50 million years ago (around 2 or 3 changes for each lineage). Taking into account how many more chromosomes birds have than mammals and the further evidence (from partial karyotypes and zoo-FISH [14]) of the apparent paucity of arrangements between macrochromosomes in all birds (even the ancient ratite birds [14]) it seems reasonable to suggest that conservation of synteny among the genomes of avian species appears to be much greater in birds than in mammals.

### Approaches for determining the molecular nature of inter-chromosomal changes

In applying three approaches to determine the molecular nature of inter-chromosomal changes we have the opportunity to corroborate the accuracy of each independently. In essence the cross species painting is limited in its resolution, even when applied to lampbrush chromosomes unless, as in this case here, when we wish to correlate the breakpoint to an identifiable chromosomal structure such as a centromere. The systematic BAC mapping is accurate but laborious and relies on all BACs working cross species (which they do only approximately 70% of the time between chicken and turkey). The use of turkey chromosome paints on to the tiling path array however was a single experiment. The potential for applying flow-sorted chromosome paints to tiling path arrays as a means of determining ancestral breakpoint and fusion points is enormous.

The results from the three approaches were not congruent for MGA9, which gave a signal only when hybridized to turkey and chicken metaphase chromosomes but not on chicken lampbrush chromosomes or on the chicken microarray. However, the chromosome paint for MGA9 gave a comparatively weak signal even on metaphase chromosomes; the relatively low quality of this paint is the most likely explanation for the failure of the lampbrush chromosome and microarray experiments.

### CNVs as revealed by array CGH

CNVs appear to be found at a lower frequency in chickens compared to human, almost one-third in number. The basis of this is unknown but may be related to the three-fold more compact chicken genome, with fewer opportunities for CNV generation. The observation that almost 50% of the CNVs in the chicken genome appear to map to the same regions as CNVs mapped from a comparison of chicken/turkey genomes suggests a common mechanism of generation. A similar observation has been made by comparing CNVs in chimp and human genomes [7]. 58 CNVs were identified from a comparison of chimp and human [7] compared to only 16 in this study. While it is possible that, when we look at several Galliform species and several individuals within each species, more inter-specific CNVs may be located it seems unlikely that there would be an order of magnitude more. Thus while chicken/turkey diverged from a common ancestor 25–30 MYA and human/chimp only 6 MYA it seems reasonable to suggest that the low karyotypic variability among birds (compared to mammals) is mirrored by a low level of CNVs also. It has been suggested that segmental duplications facilitate chromosome rearrangements and correlate with CNVs; therefore the lack of segmental duplications in the avian genome[1] might explain the low number of chromosomal rearrangements in Galliformes in particular and birds in general. Comparison of other species would shed further light on this hypothesis. Besides the generation of CNVs by non-homologous recombination or other mechanisms, CNVs may have functional consequences and may be of selective advantage. Inspection of the genomic regions at the CNVs indicates that they are associated more often than not with genes. From Table 3 it is clear that these CNV regions are also more highly conserved in avian/amphibian/fish species than mammals, suggesting a link with egg-laying species. The function of the novel genes found in these regions may shed light on this idea. An alternative to gene loss may be rapid sequence divergence in mammalian lineages. Cases of rapid divergence are supported by associations with the rapidly evolving proteins of the immune (e.g. MHC and interleukin enhancer binding factor 3) and olfactory (e.g. olfactory receptors) systems. This observation suggest a common link between avian and mammalian CNVs, where analysis of Gene Ontology [28] terms showed that genes involved in acquired immunity, innate immunity, or olfaction were also significantly overrepresented within human CNVs.

Comparative maps between vertebrate genomes suggest that rates of chromosomal change can vary widely [29]. In this study we extend these observations and provide further evidence that avian genomes are more stable both at the macro-level in terms of chromosomal rearrangements and the micro-view for CNVs. The fixation of mutations (including chromosome rearrangements and copy number variation) is a product of the rate of their generation and the rate they are fixed in a population. The molecular processes that generate such genomic changes may vary between birds and mammals. For example, mammalian genomes have more repetitive elements and segmental duplications than birds and these sequences are thought to stimulate chromosome rearrangements and copy number variation. It must be borne in mind however that, once a mutation has arisen, it must be fixed in the population either by chance or by selective advantage, eventually to contribute to the differences between species. Differences between mammals and birds at both the molecular, ecological and behavioral levels are therefore possible reasons to explain the apparent differences in the stability of their genomes.

## Conclusion

This is the first study to combine the modalities of cross-species chromosome painting, zoo-FISH of known BAC clones and array CGH to gain insight of comparative genomics between two avian species. In so doing the first insight into the conservation of microchromosomes is provided, as is the first comparative cytogenetic map of an avian species.

## Methods

### Cell culture and chromosome preparation

Chicken eggs were supplied by Hill Top Farm, Cambridgeshire, UK and Friday's Farm, Kent, UK. Turkey eggs were provided by British United Turkeys, Chester, UK. Fibroblast cultures were established from 5- to 7-day-old embryos. Chromosome preparation using mitostatic treatment with colcemid, hypotonic treatment with 75 mM KCl and fixation with 3:1 methanol:acetic acid followed standard protocols [30,31]. Chicken lampbrush chromosomes were prepared as previously described [32,33].

### Generation of chromosome paints

Chromosome paints for chicken macrochromosomes GGA1–10 and Z and turkey macrochromosomes MGA1–9 and Z were isolated by fluorescence-activated chromosome sorting at the Cambridge Resource Centre for Comparative Genomics (Cambridge Veterinary School, University of Cambridge, UK) as previously described [27,31]. Chromosome paints for chicken microchromosomes GGA11–28 were isolated by needle microdissection [27,34]. Macro- and microchromosome paints were amplified by DOP-PCR [35] and labeled by a secondary round of DOP-PCR incorporating biotin-16-dUTP or digoxigenin-11-dUTP (Roche). As described in [26] and [30], microchromosomal paints were generated from amplifications of single chromosomes at random, then distinguished from one another by dual color FISH.

### Isolation, amplification and labeling of chicken BACs

BAC clones were selected from the Wageningen chicken BAC library [36] based on the position of markers on the chicken consensus linkage map [37] and were chosen at regular intervals across the different linkage groups/chromosomes except in regions of putative evolutionary rearrangements for which a more concentrated suite of BAC clones was selected. All BACs are anchored to the chicken genome assembly by linkage and end-sequencing. BACs were extracted using the Tepnel Nucleoplex BAC Automated DNA Purification System (Tepnel Life Sciences) and labeled by nick translation with biotin-16-dUTP or digoxigenin-11-dUTP (Roche) following standard protocols.

### Fluorescent *in situ *hybridization

Slides with metaphase preparations were aged for 1 hour at 70°C on a hotplate. BACs and/or chromosome paints were applied to the metaphase preparations and sealed under a cover slip before simultaneous denaturation of probe and target DNA for 5 minutes on a 68°C hotplate. Hybridization was carried out in a humidified chamber for 24–72 hours at 37°C. Following post-hybridization washes (2 minutes in 0.4 × SSC/0.3% Igepal at 73°C; 1 minute in 2 × SSC/0.1% Igepal at RT; 15 minutes in 4 × SSC/0.05% Tween 20 at RT; 25 minutes in 4 × SSC/0.05% Tween 20/2% BSA at RT), biotin- and digoxigenin-labeled probes were detected with streptavidin-Cy3 (Amersham) and fluorescein anti-digoxigenin (Roche), respectively, and counterstained using Vectashield with DAPI (Vector Labs). Confirmation of BAC order was achieved by dual color experiments where biotin- and digoxigenin-labeled probes were hybridized simultaneously.

### Image capturing and analysis

Images were captured using a Leica epifluorescence microscope equipped with cooled CCD camera (Photometrics) and Smart Capture software (Digital Scientific). Signal position on chromosomes was established by measuring the fractional length from the p-terminus (FLpter) [38]. The signal positions were measured as Analysis was performed using GIMP, the freeware GNU Image Manipulation Program following the protocol described by [38]. The total length of the chromosome with the BAC signal and the length from the signal to the p terminus were measured using the measure tool and were expressed as a ratio, with the p terminus being 0.0 and the q terminus being 1.0. At least 10 metaphases were analyzed for each BAC.

### Analysis of sequence composition of chromosome arms

All data used the WUSTL 2.1 (May 2006) build of the chicken genome. Gene position and gene structure are derived from the ENSEMBL 43 gene build and downloaded through BioMart. The CpG and repeats information have been retrieved from the UCSC Genome Browser  for the corresponding gene build. CpG islands have been identified using an algorithm written by Andy Law with minor modifications by Angie Hinrichs [1]. The repeats data have been identified with Arian Smit's RepeatMasker program [39], which screens DNA sequences for interspersed repeats and low complexity DNA sequences. RepeatMasker uses the RepBase library of repeats from the Genetic Information Research Institute [40]. Only the repeated regions have been considered, by filtering out the low complexity one. The corresponding number of nucleotides involved in such structures has been divided by the length of each arm. The genomic GC% has been calculated with 50 kb windows. The GC% for introns and exons has been calculated for each intron and exon. For the graphical representation, a window of 1 Mb size has been used for each represented parameter. For the gene density, it corresponds to the cumulative plot of uniform distribution subtracted to the real gene distribution across the chromosome. Same has been used for the representation of the compactness (exons length/(introns + exons length)) and CpG islands (cumulative number of CpG). On each graph, the centromeric region has been identified.

### Comparative Genome Hybridization (CGH) experiments

The NimbleGen chicken whole-genome tiling array (Catalogue Number/Design Name B3791001-00-01, galGal3 WG CGH – Roche NimbleGen, Milton Keynes, UK) was used for all microarray experiments. It contains 385,000 50-mer oligonucleotides with an average spacing of 2,586 base pairs (source – UCSC, build – galGal3) and was interrogated with chromosome paints for turkey chromosomes 3, 4, 6 and 9 and, for the array CGH experiments, with chicken and turkey whole genomic DNA. Turkey chromosome paints were generated as described above but not labeled, and whole genomic DNA was extracted in house; the reference (Red Jungle Fowl) DNA for the array CGH experiments was a gift from Dr Hans Cheng (Michigan State University). Labeling of chromosome paints and genomic DNA and hybridization to the NimbleGen array were performed by the company (NimbleGen) and used a random prime labeling kit to incorporate modified nucleotides by either amino-allyl or direct linkage to either of the two dyes used (Cy3 and Cy5). All of the hybridizations in this experiment used two dyes per slide (Cy3 and Cy5). For the array chromosome painting experiments, MGA3 (Cy3) was co-hybridized with MGA6 (Cy5) and MGA9 (Cy3) co-hybridized with MGA4 (Cy5). For the array CGH Red Jungle Fowl DNA (reference, Cy5) was co-hybridized with either chicken or turkey test DNA (Cy3).

CGH analysis proceeded in three stages, normalization, window averaging and segmentation. After combining the signal intensity and genomic coordinate information, the Cy3 and Cy5 signal intensities were normalized to one another using *Qspline *normalization [41]. *Qspline *is a robust non-linear method for normalization using array signal distribution analysis and cubic splines. Once normalized, the data was prepared for DNA segmentation analysis. This included a window averaging step, where the probes that fall into a defined base pair window size are averaged, using the *Tukey's biweight *mean [42]. The *Tukey's biweight *method yields a robust weighted mean that is relatively insensitive to outliers, even when extreme. A new position was assigned to this average, which is the midpoint of the window. Window sizes of 30, 60 and 150 kb were used. The *circular binary segmentation *algorithm [43] was used to segment the averaged log2 ratio data. DNA segments were called by attempting to break the segments into sub-segments by looking at the t-statistic of the means. Permutations (n = 1000) were used to provide the reference distribution. If the resulting p-value was below the threshold (default of p = 0.01), then a breakpoint was called. A pruning step was used to remove spurious segments, rejecting segments where the standard deviation of the means was not sufficiently different. By default, a cut off of 1.5 standard deviations was used. There are three kinds of output produced by the DNA segmentation analysis: a segmentation table, a PDF plot, and a GFF file. The PDF plot and GFF file show the log2 ratios as black dots and the predicted segments as red lines. The segmentation table shows the endpoints and mean log2 value of each predicted segment. This is the data used to draw the lines on the individual plots using the SignalMap™ viewer (NimbleGen, version 1.8). Information regarding GFF file formats can be found on the Wellcome Trust Sanger Institute web site [44].

## Authors' contributions

DKG co-conceived and supervised the project and drafted the manuscript. He was the principal investigator on the BBSRC grant that funded the project and supervisor of LBR, HGT, MV and JSM. LBR, HGT, JSM, AV and VF performed and collated the zoo-FISH experiments. RPMAC and MAMG isolated, genetically mapped and provided the BAC clones. SD and EG isolated the lampbrush chromosomes and performed the FISH experiments on them. RT performed the bioinformatic analysis, arranged the microarray experiments and performed the data analysis on them. WC and DW performed the sequence analysis. MV made significant contributions to the FISH, manuscript completion and data analysis. DWB co-conceived the project and was the co-investigator on the BBSRC grant that funded the project and performed further data analysis on the microarrays.
